# Development and validation of prediction models for hypertension risks: A cross-sectional study based on 4,287,407 participants

**DOI:** 10.3389/fcvm.2022.928948

**Published:** 2022-09-26

**Authors:** Weidong Ji, Yushan Zhang, Yinlin Cheng, Yushan Wang, Yi Zhou

**Affiliations:** ^1^Department of Medical Information, Zhongshan School of Medicine, Sun Yat-sen University, Guangzhou, China; ^2^Department of Maternal and Child Health, School of Public Health, Sun Yat-sen University, Guangzhou, China; ^3^Center of Health Management, The First Affiliated Hospital of Xinjiang Medical University, Urumqi, China

**Keywords:** hypertension, machine learning, prediction model, classifier, LASSO

## Abstract

**Objective:**

To develop an optimal screening model to identify the individuals with a high risk of hypertension in China by comparing tree-based machine learning models, such as classification and regression tree, random forest, adaboost with a decision tree, extreme gradient boosting decision tree, and other machine learning models like an artificial neural network, naive Bayes, and traditional logistic regression models.

**Methods:**

A total of 4,287,407 adults participating in the national physical examination were included in the study. Features were selected using the least absolute shrinkage and selection operator regression. The Borderline synthetic minority over-sampling technique was used for data balance. Non-laboratory and semi-laboratory analyses were carried out in combination with the selected features. The tree-based machine learning models, other machine learning models, and traditional logistic regression models were constructed to identify individuals with hypertension, respectively. Top features selected using the best algorithm and the corresponding variable importance score were visualized.

**Results:**

A total of 24 variables were finally included for analyses after the least absolute shrinkage and selection operator regression model. The sample size of hypertensive patients in the training set was expanded from 689,025 to 2,312,160 using the borderline synthetic minority over-sampling technique algorithm. The extreme gradient boosting decision tree algorithm showed the best results (area under the receiver operating characteristic curve of non-laboratory: 0.893 and area under the receiver operating characteristic curve of semi-laboratory: 0.894). This study found that age, systolic blood pressure, waist circumference, diastolic blood pressure, albumin, drinking frequency, electrocardiogram, ethnicity (uyghur, hui, and other), body mass index, sex (female), exercise frequency, diabetes mellitus, and total bilirubin are important factors reflecting hypertension. Besides, some algorithms included in the semi-laboratory analyses showed less improvement in the predictive performance compared to the non-laboratory analyses.

**Conclusion:**

Using multiple methods, a more significant prediction model can be built, which discovers risk factors and provides new insights into the prediction and prevention of hypertension.

## Introduction

Nowadays, hypertension has affected 1.13 billion people worldwide (1). It exacerbates the burden of stroke, ischemic heart disease, other vascular diseases, and kidney disease (2). The number of people with hypertension worldwide exceeded 1 billion in 2019, which was doubled since 1990 (3). In China, the proportion of adults with hypertension has increased substantially over the past 40 years, and people's awareness regarding hypertension, the diagnosis, treatment, and control rates of hypertension are low, especially in the western region (4, 5). Therefore, it is of vital importance to strengthen the pre-screening of hypertension and carry out preventive intervention and treatment for high-risk and potential groups (6).

The prediction model has been proven to be an effective and economical tool to identify individuals with a high risk of hypertension (7). However, many studies have confirmed that the risk prediction models developed for one population cannot be effectively applied to other populations (8–11). Although some hypertension risk prediction models have been established in China in the past 10 years (12–16), there were some disadvantages, such as small sample size and lack of important features (ethnicity and poor prediction effect), which limits the generalizability of models. Therefore, it is urgent to establish a hypertension prediction model with a good prediction effect and strong generalizability in China.

Machine learning (ML) is a collection of techniques that automatically learn features from data and do not require the data structure, and mainly includes classification and regression tree (CART), random forest (RF), extreme gradient boosting decision tree (XGBoost), naive Bayes (NB), and artificial neural network (ANN). ML shows an excellent performance in disease prediction in recent years (17, 18). The application of ML algorithms to predict hypertension can provide some new ideas for understanding the pathophysiological mechanisms underlying hypertension and for exploring therapeutic targets. However, some studies showed that the incremental predictive performance beyond standard methods might be limited (19–21), while others showed that there were no advantages of ML over classical statistical models, such as logistic regression (LR) (22, 23). In the aspect of hypertension prediction, most studies only test the predictive performance of ML models or LR models alone, without conducting comparative studies (13, 16, 24–26). Therefore, it is unclear whether the ML method is better than traditional classical statistical models in the prediction of potential hypertension populations.

Currently, no studies investigated the predictive ability of the semi-laboratory analyses and the non-laboratory analyses. Therefore, in this study, we constructed and compared the tree-based ML models, such as CART, RF, adaboost with decision tree (ADABoost), XGBoost, other ML models, such as NB and ANN, and traditional LR models based on non-laboratory and semi-laboratory analyses, respectively, aiming to develop optimal hypertension screening model for large populations. As we know, the hypertension screening model presented in this study is the first to be established by comparing various algorithms systematically and comprehensively with multi-ethnic and large samples.

## Methods

### Study population

The national physical examination (NPE) is a free physical examination provided by the Chinese government for all Xinjiang people. Epidemiologists and medical staff at Xinjiang Uygur Autonomous Region Center for Disease Control and Prevention have designed a standard physical examination form, which mainly consisted of a questionnaire survey, routine examinations, and laboratory tests in three parts. All examinations were conducted by a professional medical team with medical qualifications and fieldwork experience. All participants were required to take their unique identity document (ID) card, which was used as the only proof of identity.

All data were aggregated to the Health Management Hospital of Xinjiang Medical University. For routine examination, the items included standing height, weight, waist circumference (WC), heart rate (HR), blood pressure, and abdominal ultrasound. In addition, three 10 ml samples of non-fasting blood samples were collected into vacuum tubes, and then the samples were kept in a portable insulated cold box with ice packs and taken to a local research laboratory for immediate processing. Blood test indicators contained blood sugar and blood biochemistry.

The data in this study were collected from the NPE project, and a total of 4,336,239 people who had signed informed consent forms were included. The excluded criteria were (i) age < 18 years and (ii) the data missing rate > 20%. A total of 4,287,407 participants from 14 regions in Xinjiang Province were finally included in this study for further analysis after strict screening procedures. Detailed population distributions were as follows: Hotan (662,643), Ili (614,468), Aksu (590,630), Changji (339,019), Tacheng (266,494), Bayingolin Mongolia (206,897), Altay (184,948), Turpan (154,105), Bortala Mongolia (86,864), Hami (83,560), Kizilsu Kirgiz (82,078), Karamay (271), Kashgar (622,610), and Urumqi (392,820).

**Figure 1:**
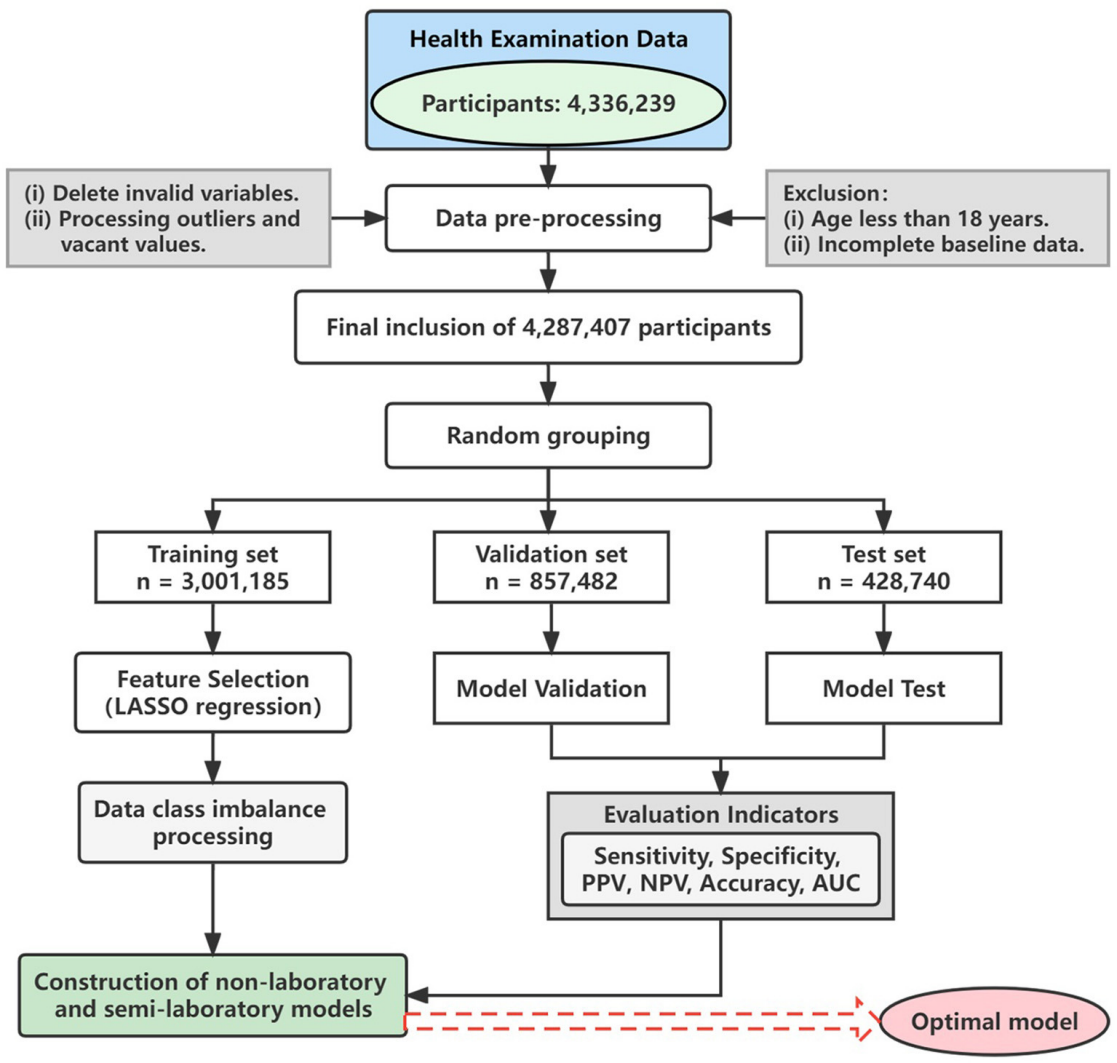
Flow chart. LASSO, least absolute shrinkage and selection operator; PPV, positive predictive value; NPV, negative predictive value; AUC, area under the receiver operating characteristic curve.

Furthermore, nearly 200 variables irrelevant to this study were deleted, such as names, home addresses, and contact numbers, and then the missing and extreme values of the remaining variables were processed. Continuous variables were imputed by means, while categorical variables were imputed by mode. Figure 1 shows the detailed analysis process. This study was conducted in accordance with the principles outlined in the “Helsinki Declaration” and was approved by the Ethics Committee and Institutional Review Committee of the Xinjiang Uygur Autonomous Region Center for Disease Control and Prevention.

### Definition of hypertension

Hypertension patients met the following criteria: systolic blood pressure (SBP) ≥ 140 mmHg and diastolic blood pressure (DBP) ≥ 90 mmHg in the absence of antihypertensive drugs, or someone with a hypertension history, though the blood pressure did not reach the above level when undertaking antihypertensive drugs.

### Predictors considered

With the characteristics of large data size, multiple variables, and the existence of many outliers and gaps, pre-processing of the data is important. In total 30 variables from the three components were used to construct the predictive model and evaluate the potential risk factors of hypertension. Variables are listed in Table 1.

**Table 1:** Information description of included variables.

**Data sources**	**Variable**	**Variable type**
Questionnaire	Age	Continuous variable
	Sex	Categorical variable (“male” or “female”)
	Ethnicity	Categorical variable (“han,” “uyghur,” “kazakh,” “hui,” “other ethnic groups”)
	EF	Categorical variable (“not exercising,” “occasionally,” “more than once a week,” “daily”)
	SS	Categorical variable (“never smoked,” “smoking,” “quit smoking”)
	DF	Categorical variable (“never,” “occasionall,” “often,” “every day”)
	DM	Categorical variable (yes or no)
	PH	Categorical variable (yes or no)
Routine examination	Hight	Continuous variable
	Weight	Continuous variable
	BMI	Continuous variable
	SBP	Continuous variable
	DBP	Continuous variable
	WC	Continuous variable
	ECG	Categorical variable (normal or abnormal)
	HR	Categorical variable (normal or abnormal)
Laboratory test	HGB	Continuous variable
	WBC	Continuous variable
	PLT	Continuous variable
	FBG	Continuous variable
	SGPT	Continuous variable
	SGOT	Continuous variable
	ALB	Continuous variable
	TBIL	Continuous variable
	SCR	Continuous variable
	BUN	Continuous variable
	TC	Continuous variable
	TG	Continuous variable
	LDLC	Continuous variable
	HDLC	Continuous variable

Height was measured to the nearest 0.5 cm and weight to the nearest 0.1 kg. The height-weight scale had been calibrated before using. Light clothes and no shoes were required for the participants. BMI was calculated as weight (Kg)/height2 (m2). EF, exercise frequency; SS, smoking status; DF, drinking frequency; DM, diabetes mellitus; PH, parental hypertension; BMI, body mass index; SBP, systolic blood pressure; DBP, diastolic blood pressure; WC, waist circumference; ECG, electrocardiogram; HR, heart rate; HGB, hemoglobin; WBC, white blood cell; PLT, platelet; FBG, fasting blood glucose; SGPT, serum glutamic-pyruvic transaminase; SGOT, serum glutamic-oxaloacetic transaminase; ALB, albumin; TBIL, total bilirubin; SCR, serum creatinine; BUN, blood urea nitrogen; TC, total cholesterol; TG, triglyceride; LDLC, low-density lipoprotein cholesterol; HDLC, high-density lipoprotein cholesterol.

### Statistical analysis

Continuous variables were expressed as median (IQR: inter-quartile range), and categorical variables were expressed as counts (percentage). Variables were compared between hypertension and non-hypertension groups. The *t*-test or Mann–Whitney test was used for continuous variables, while for categorical variables, the chi-square test or Fisher's exact test was used. Statistical significance was inferred at a two-tailed *P*-value<0.05.

#### Grouping and feature selection

The population was randomly divided into the training set (3,001,185), the validation set (857,482), and the test set (428,740), with a ratio of 7: 2: 1. Then, the least absolute shrinkage and selection operator (LASSO) regression were used to select the variables in the training set (27). LASSO regression was characterized by variable selection and regularization while fitting a generalized linear model, which was suitable for continuous, binary, and multivariate discrete variables.

#### Data imbalance processing

In this study, the number of non-hypertension participants was larger than the hypertension participants, which indicates that the sample size was imbalanced, while minority classes were harder to predict using ML methods (28, 29). An over-sampling technique, that is, borderline synthetic minority over-sampling technique (Borderline-SMOTE), was used to deal with the negative influence due to the imbalanced classification problem.

The synthetic minority over-sampling technique (SMOTE) was introduced by Chawla et al. (30), as a way to deal with the minority classes in a dataset. The fundamental idea of this algorithm is to analyze and simulate, and add the new sample simulated artificially into the original dataset to balance the classes in the original data. But there were two obvious shortcomings of SMOTE: (1) prone to sample overlap and (2) the attribute characteristics and the distribution characteristics of adjacent samples are not considered. Therefore, many adaptive sampling methods are developed to solve the above limitations, among which the Borderline-SMOTE algorithm is the most representative one (31).

Borderline-SMOTE is an advanced over-sampling algorithm based on SMOTE, which uses minority class samples on the boundary to synthesize new samples, and therefore improves the class distribution of the samples. In Borderline-SMOTE sampling, the minority class samples are divided into three categories: safe, danger, and noise. Safe means more than half of the surrounding samples are minority class samples. Danger means that more than half of the surrounding samples are majority class samples, which are regarded as boundary samples. Besides, noise refers to the majority class of samples around the sample, which is regarded as noise. Finally, only the minority class samples that behave as danger are over-sampled.

#### Variable coding

The preprocessing.LabelEncoder algorithm of sklearn.preprocessing library in python software was used to digitize the labels, and preprocessing.OrdinalEncoder algorithm was used to digitize the orderly categorical variables of characteristics. The preprocessing.OneHotEncoder algorithm was used to convert the nominal variables to the dummy variables.

#### Prediction models

This study established three kinds of hypertension predictive models, including tree-based ML models (CART, RF, ADABoost, and XGBoost), other ML models (ANN and NB), and traditional LR models. On the basis of the above models, we analyzed non-laboratory and semi-laboratory features separately depending on whether blood test data were included or not.

The CART algorithm is based on tree arrangement and describes the classification process depending on input features. There were some advantages of CART, such as fast computing, high accuracy, no requirement of domain knowledge or parametric assumptions, and suitable for high-dimensional data, but it has some shortcomings, such as high variance and over-fitting phenomenon, which limits its practicality as an independent predictive model. RF is an algorithm that combines bagged ensemble learning theory with random subspace methods (32), aiming at constructing many independent evaluators and then selecting the results supported by most evaluators or choosing the mean values. ADABoost and XGBoost algorithms (33) aim at combining the power of the weak evaluator to predict the hard-to-evaluate samples repeatedly, in order to construct a strong evaluator.

The ANN is a computing system based on human brain neurons (34). ANN can deal with the interactions between complex and non-linear variables. ANN consists of a multi-hidden layer neural network and a single hidden layer neural network. Each layer contains some neurons connected by directed arcs with variable weights. In this study, the neural network contains three layers: the input layer accepts all risk factors, the hidden layer processes the information, and the output layer calculates the response. NB is a classical ML algorithm, which calculates the probabilities of each attribute by applying Bayes' rule and predicts the class based on the highest prior probability (35).

The LR is a generalized linear regression analysis model, and aims to find out the best fitting model to describe the relationship between the dependent variables and independent predictors (36). This model was most extensively applied because of the good effect of disease predictions.

#### Model evaluation

To optimize the model effect, we adjust the parameters of each model based on the learning curve and grid search, so as to find the optimal combination of parameters. Besides, we calculated the sensitivity, specificity, positive predictive value (PPV), negative predictive value (NPV), accuracy, Youden index, and area under the receiver operating characteristic (AUC) curve of each model based on the confusion matrix to evaluate the pros and cons.

#### Feature importance ranking

According to the results of the LR model, the absolute values of the regression model Z statistic (23, 37) were calculated and adjusted the sum to 1 (the higher the value, the greater the effect on hypertension). Then, the feature importance ranking plot of the LR model was drawn.

Machine Learning algorithms can also measure the importance of different features. Different from the odds ratio (OR) of the regression model, the machine algorithm cannot evaluate a simple explanatory value because the relationship fitted by the machine algorithm is complex. Therefore, the relationship is usually not directly generalized to any one parameter, and there is no causal relationship, not even a statistical explanation (18). This measure is usually viewed as the sorting of how important each variable is to the model fit, which is a method to generate hypotheses in order to identify the factors requiring further study and also provides insight into the factors having the greatest impact on predictions. Therefore, a feature importance ranking plot was drawn for the ML algorithm which showed the best prediction.

All analyses were carried out with the python 3.8.3 version. Null and outlier determination and interpolation were performed by the “Pandas” library, “NumPy” library, and “Matplotlib” library. Data imbalance was solved by the “Imlearn” library, and build and validate ML models by the “Sklearn” library. LASSO penalized LR was performed by the “Glmnet” package of the R software 4.1.0 version.

## Results

### Gender and age differences in hypertension

After pre-processing of data, 4,287,407 people were left, consisting of 2,009,970 men (46.9%) and 2,277,437 women (53.1%). From Table 2, we can observe that the prevalence of hypertension was 22.1% in men and 23.7% in women, and the prevalence of hypertension was higher in women than in men (*P* < 0.001). This study further analyzed the differences in the prevalence of hypertension in two genders with different age groups, and we found that in the 18–29 age group and 30–45 age group, men had a higher prevalence (*P* < 0.001), while in the 46–65 age group and over 65 age group, women had a higher prevalence (*P* < 0.001). The prevalence of hypertension increases sharply with age in both genders.

**Table 2:** Differences in the prevalence of hypertension between men and women in this study (*N* = 4,287,407).

**Variables**	**Total**	**Non–Hypertensive**	**Hypertension**	**Prevalence of hypertension**	**P-value**
**Sex**, ***n*****(%)**					<0.001
Female	2,277,437 (53.1)	1,736,267 (52.6)	541,170 (54.9)	23.7	
Male	2,009,970 (46.9)	1,565,709 (47.4)	444,261 (45.1)	22.1	
**Age group**, ***n*****(%)**					
18–29					<0.001
Female	273,841 (52.6)	273,391 (52.7)	450 (29.4)	0.2	
Male	246,490 (47.4)	245,411 (47.3)	1,079 (70.6)	0.4	
30–45					<0.001
Female	560,689 (53.9)	538,282 (54.3)	22,407 (46.3)	4.0	
Male	479,894 (46.1)	453,897 (45.7)	25,997 (53.7)	5.4	
46–65					<0.001
Female	1,054,532 (53.6)	755,022 (52.5)	299,510 (56.4)	28.4	
Male	913,697 (46.4)	682,213 (47.5)	231,484 (43.6)	25.3	
65 over					<0.001
Female	388,375 (51.2)	169,572 (47.9)	218,803 (54.1)	56.3	
Male	369,889 (48.8)	184,188 (52.1)	185,701 (45.9)	50.2	

### Basic characteristics

The general characteristics of participants in this study are shown in Table 3. A total of 985,431 patients with hypertension were recruited. Compared with non-hypertension people, the median values of age, SBP, DBP, body mass index (BMI), WC, hemoglobin (HGB), white blood cell (WBC), fasting blood glucose (FBG), serum glutamic-pyruvic transaminase (SGPT), serum glutamic oxaloacetic transaminase (SGOT), serum creatinine (SCR), blood urea nitrogen (BUN), total cholesterol (TC), triglyceride (TG), and low-density lipoprotein cholesterol (LDLC) were higher in people with hypertension, and the latter are more likely to have parental hypertension (PH) and diabetes mellitus (DM). On the contrary, higher platelet (PLT) and higher albumin (ALB) levels were more common in participants without hypertension.

**Table 3:** Characteristics of participants in this study.

**Characteristics**	**Total** **(*n* = 4,287,407)**	**Non–Hypertensive** **(*n* = 3,301,976)**	**Hypertension** **(*n* = 985,431)**	***P*** **–value**
Age (median [IQR])	50.00 [38.00, 61.00]	47.00 [34.00, 55.00]	62.00 [54.00, 70.00]	<0.001
Sex (%)				<0.001
Female	2,277,437 (53.1)	1,736,267 (52.6)	541,170 (54.9)	
Male	2,009,970 (46.9)	1,565,709 (47.4)	444,261 (45.1)	
Ethnicity (%)				<0.001
Han	1,255,170 (29.3)	983,889 (29.8)	271,281 (27.5)	
Hui	198,028 (4.6)	151,551 (4.6)	46,477 (4.7)	
Kazakh	408,666 (9.5)	307,380 (9.3)	101,286 (10.3)	
Other nationalities	138,776 (3.2)	109,309 (3.3)	29,467 (3.0)	
Uyghur	2,286,767 (53.3)	1,749,847 (53.0)	536,920 (54.5)	
EF (%)				<0.001
Daily	301,849 (7.0)	197,356 (6.0)	104,493 (10.6)	
More than once a week	91,594 (2.1)	61,264 (1.9)	30,330 (3.1)	
Not exercising	3,751,779 (87.5)	2,945,712 (89.2)	806,067 (81.8)	
Occasionally	142,185 (3.3)	97,644 (3.0)	44,541 (4.5)	
SS (%)				<0.001
Never smoked	3,820,572 (89.1)	2,922,174 (88.5)	898,398 (91.2)	
Quit smoking	29,746 (0.7)	19,743 (0.6)	10,003 (1.0)	
Smoking	437,089 (10.2)	360,059 (10.9)	77,030 (7.8)	
DF (%)				<0.001
Every day	5,235 (0.1)	3,626 (0.1)	1,609 (0.2)	
Never	3,964,939 (92.5)	3,036,309 (92.0)	928,630 (94.2)	
Occasionall	293,659 (6.8)	242,935 (7.4)	50,724 (5.1)	
Often	23,574 (0.5)	19,106 (0.6)	4,468 (0.5)	
PH (%)				<0.001
No	4,085,273 (95.3)	3,169,978 (96.0)	915,295 (92.9)	
Yes	202,134 (4.7)	131,998 (4.0)	70,136 (7.1)	
DM (%)				<0.001
No	4,003,394 (93.4)	3,195,815 (96.8)	807,579 (82.0)	
Yes	284,013 (6.6)	106,161 (3.2)	177,852 (18.0)	
SBP (median [IQR])	120.00 [110.00, 130.00]	120.00 [110.00, 126.00]	126.00 [119.58, 140.00]	<0.001
DBP (median [IQR])	72.00 [67.00, 80.00]	70.00 [65.00, 80.00]	80.00 [70.00, 90.00]	<0.001
BMI (median [IQR])	24.80 [22.32, 27.44]	24.29 [22.03, 26.93]	26.06 [23.83, 29.00]	<0.001
Wc (median [IQR])	86.00 [79.00, 95.00]	85.00 [78.00, 92.00]	91.00 [83.00, 100.00]	<0.001
HR (%)				<0.001
Abnormal	39345 (0.9)	28326 (0.9)	11019 (1.1)	
Normal	4248062 (99.1)	3273650 (99.1)	974412 (98.9)	
ECG (%)				<0.001
Abnormal	895654 (20.9)	607733 (18.4)	287921 (29.2)	
Normal	3391753 (79.1)	2694243 (81.6)	697510 (70.8)	
HGB (median [IQR])	141.00 [129.00, 153.00]	140.70 [128.00, 153.00]	143.00 [132.00, 153.00]	<0.001
WBC (median [IQR])	6.20 [5.25, 7.27]	6.15 [5.20, 7.20]	6.37 [5.36, 7.45]	<0.001
PLT (median [IQR])	236.00 [198.00, 276.00]	236.00 [199.00, 276.00]	235.00 [196.00, 276.00]	<0.001
FBG (median [IQR])	5.23 [4.74, 5.74]	5.20 [4.69, 5.63]	5.43 [4.94, 6.10]	<0.001
SGPT (median [IQR])	20.90 [15.00, 28.70]	20.90 [15.00, 28.90]	21.00 [15.00, 28.30]	<0.001
SGOT (median [IQR])	21.60 [17.40, 26.70]	21.50 [17.30, 26.60]	21.80 [17.60, 27.00]	<0.001
ALB (median [IQR])	14.15 [14.15, 14.15]	14.15 [14.15, 14.15]	14.15 [14.15, 14.15]	<0.001
TBIL (median [IQR])	12.71 [9.56, 15.17]	12.71 [9.52, 15.20]	12.71 [9.60, 15.02]	0.008
SCR (median [IQR])	65.50 [54.95, 78.00]	65.20 [54.60, 77.60]	66.40 [55.56, 78.70]	<0.001
BUN (median [IQR])	4.95 [3.98, 5.99]	4.89 [3.92, 5.90]	5.11 [4.16, 6.21]	<0.001
TC (median [IQR])	4.40 [3.76, 5.10]	4.32 [3.70, 5.01]	4.60 [3.99, 5.30]	<0.001
TG (median [IQR])	1.22 [0.89, 1.68]	1.20 [0.85, 1.61]	1.34 [1.00, 1.89]	<0.001
LDLC (median [IQR])	2.46 [1.95, 3.04]	2.41 [1.92, 3.00]	2.57 [2.03, 3.20]	<0.001
HDLC (median [IQR])	1.33 [1.10, 1.64]	1.33 [1.10, 1.64]	1.32 [1.10, 1.63]	<0.001

For continuous variables, the data were expressed as median [IQR: inter–quartile range], and for categorical variables, the data were expressed as counts (percentage). EF, exercise frequency; SS, smoking status; DF, drinking frequency; DM, diabetes mellitus; PH, parental hypertension; BMI, body mass index; SBP, systolic blood pressure; DBP, diastolic blood pressure; WC, waist circumference; ECG, electrocardiogram; HR, heart rate; HGB, hemoglobin; WBC, white blood cell; PLT, platelet; FBG, fasting blood glucose; SGPT, serum glutamic–pyruvic transaminase; SGOT, serum glutamic–oxaloacetic transaminase; ALB, albumin; TBIL, total bilirubin; SCR, serum creatinine; BUN, blood urea nitrogen; TC, total cholesterol; TG, triglyceride; LDLC, low–density lipoprotein cholesterol; HDLC, high–density lipoprotein cholesterol.

### Features extraction

**Figure 2:**
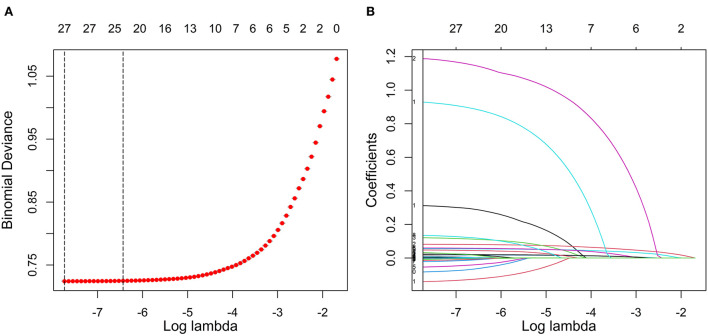
Feature selection using LASSO regression in the training set. **(A)** Cross-validation was performed 10 times to select the optimal parameters (lambda) of the LASSO model. **(B)** LASSO coefficient profile of 24 characteristics. In the LASSO algorithm, with the change of lambda, the trajectory of each hypertension-related characteristic coefficient is observed in the LASSO coefficient profile. LASSO, least absolute shrinkage and selection operator.

In this study, the LASSO regression model was used to select the features of the training set data. The results show that there were 24 variables with non-zero coefficients in the LASSO regression model (Figure 2), including sex, age, ethnicity, SBP, DBP, BMI, WC, exercise frequency (EF), drinking frequency (DF), PH, DM, HGB, WBC, PLT, FBG, electrocardiogram (ECG), SGOT, ALB, total bilirubin (TBIL), BUN, TC, TG, LDLC, and high-density lipoprotein cholesterol (HDLC). These 24 variables were used in three types of hypertension prediction models.

### Class balance

The sample size of hypertensive patients in the training set was expanded to 2,312,160 by the Borderline-SMOTE algorithm, and finally, 4,624,320 non-hypertensive and hypertensive samples were obtained (Table 4).

**Table 4:** Borderline–SMOTE over–sampling balanced dataset description.

**Dataset**	**Non–Hypertensive/Hypertensive**	**Ratio**	**Description**
Training set data	2,312,160/689,025	3.36:1	Original data with full instances
Borderline–SMOTE data	2,312,160/2,312,160	1:1	Dataset is balanced utilizing Borderline–SMOTE oversampling

### Tuning of parameters

**Figure 3:**
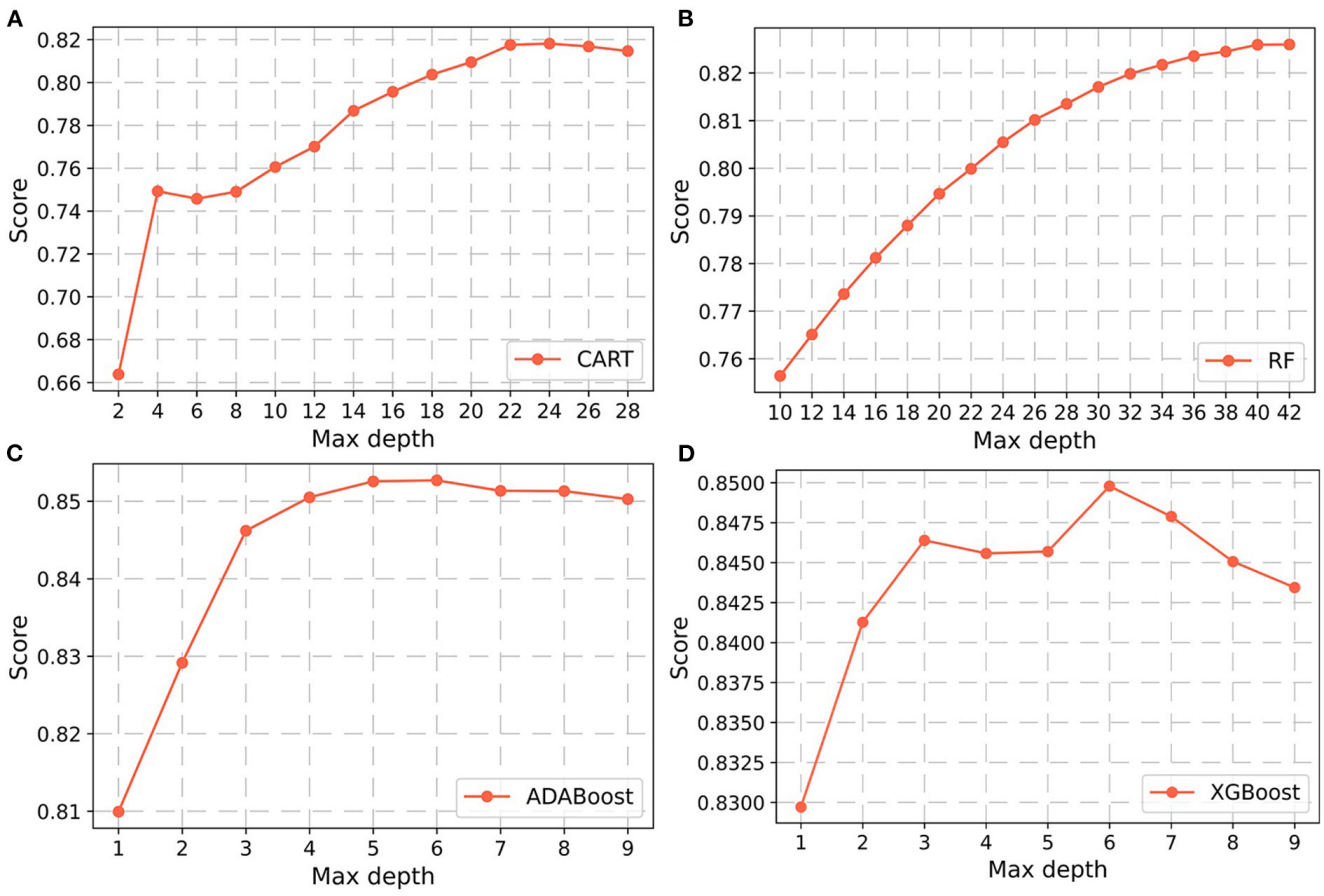
Parameter selection for four non-laboratory prediction models. Using the learning curves for **(A)** CART. **(B)** RF. **(C)** ADABoost, and **(D)** XGBoost respectively, the scores (accuracy) of each algorithm at different tree depths are shown in Figure. Abbreviations: CART, classification and regression tree; RF, random forest; ADABoost, adaboost with decision tree; XGBoost, extreme gradient boosting decision tree.

**Figure 4:**
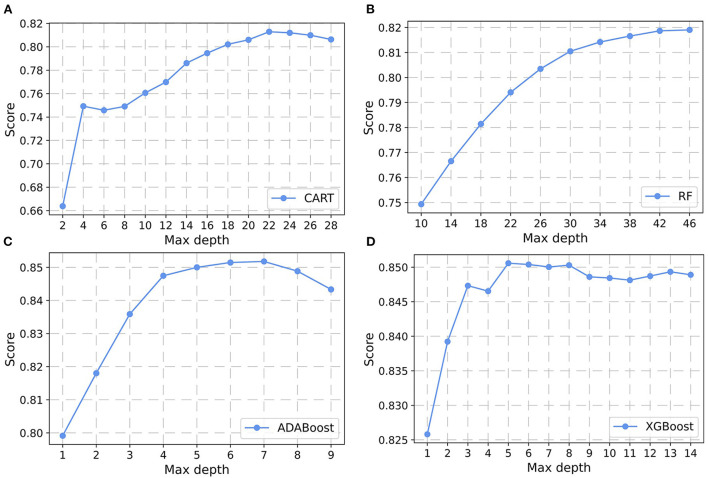
Parameter selection for four semi-laboratory prediction models. Using the learning curves for **(A)** CART. **(B)** RF. **(C)** ADABoost, and **(D)** XGBoost, respectively, the scores (accuracy) of each algorithm at different tree depths are shown in Figure. CART, classification and regression tree; RF, random forest; ADABoost, adaboost with decision tree; XGBoost, extreme gradient boosting decision tree.

In the non-laboratory and semi-laboratory analyses, we optimally adjusted the training set parameters of the four “tree” models, and listed the score (accuracy) of each parameter under the different models in the validation set. The results showed that, on the basis of the optimization of the other parameters, the “tree” depths of CART, RF, ADABoost, and XGBoost in the non-laboratory analyses were 24, 40, 5, and 6, respectively (Figure 3) while in the semi-laboratory analyses were 22, 44, 7, and 5, respectively (Figure 4). Thus, a relatively economical and accurate classification tree model is obtained, respectively.

### Comparison of model performance

**Figure 5:**
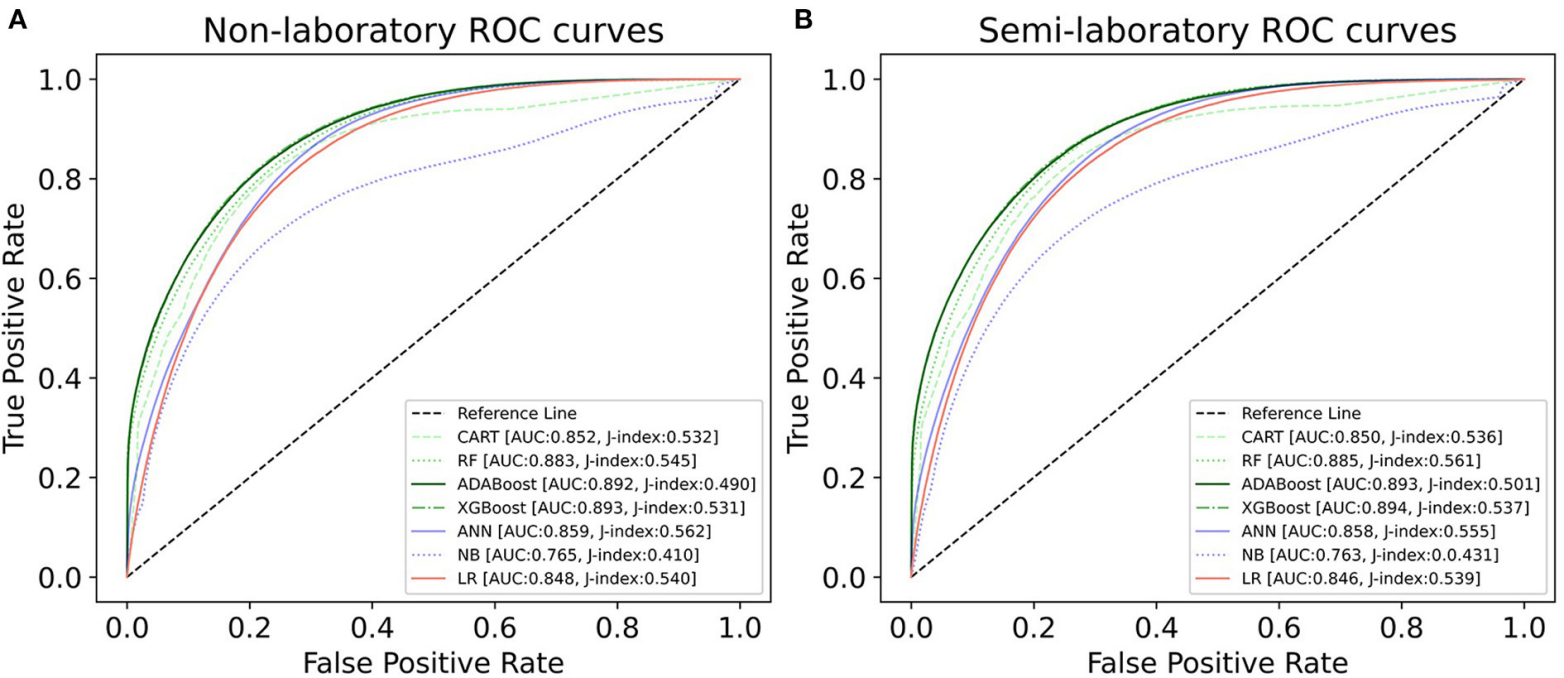
ROC curves for each classification algorithm for the non-laboratory and semi-laboratory models. **(A)** Non-laboratory model. **(B)** Semi-laboratory model. Youden's J-index combines sensitivity and specificity into a single measure (sensitivity + specificity−1) and has a value between 0 and 1. In a perfect test, Youden's index equals 1. ROC, receiver operating characteristic; CART, classification and regression tree; RF, random forest; ADABoost, adaboost with decision tree; XGBoost, extreme gradient boosting decision tree; ANN, artificial neural network; NB, naive Bayes; LR, logistic regression; AUC, the area under the ROC curve.

We constructed three classification models of tree-based ML models (CART, RF, ADABoost, and XGBoost), other ML models (ANN and NB), and traditional classical models (LR) in this study. Supplementary Tables S1, S2 presented the algorithm performances of non-laboratory and semi-laboratory analyses in the validation set, respectively. Tables 5, 6 presented the algorithm performances of non-laboratory and semi-laboratory analyses in the test set, respectively. The heat map showed the confusion matrix, where the larger the value, the darker the color of the area, i.e., the color of the TN and TP areas were closer to red or blue. On the contrary, the lighter the color of the FN and FP regions, the higher the accuracy of the classification model. XGBoost algorithm had a great performance in predicting the risk of hypertension in a large population of China, whose AUC of non-laboratory and semi-laboratory was 0.893 and 0.894, respectively. The NB algorithm was less effective in predicting hypertension. Some of the algorithms (RF, ADABoost, and XGBoost) in the semi-laboratory analysis incorporating blood test data showed little improvement in predictive performance compared to the non-laboratory analysis. Supplementary Figures S1, S2, and Figure 5 show the receiver operating characteristic (ROC) curve of all classifiers.

**Table 5:** Performance of each algorithm in the test set for non–laboratory analysis (*n* = 428,740).

**Models**	**Sub–Algorithms**	**Confusion matrix**	**Sensitivity**	**Specificity**	**PPV**	**NPV**	**Accuracy**	**AUC**
Tree–based ML models	CART	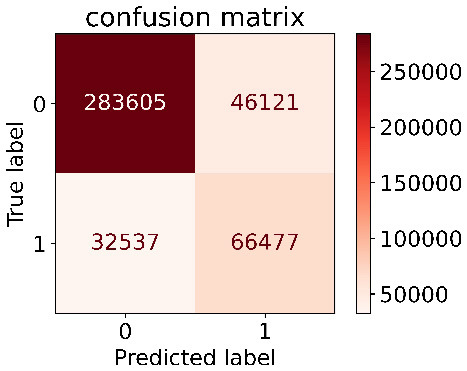	0.671	0.860	0.590	0.897	0.817	0.852
	RF	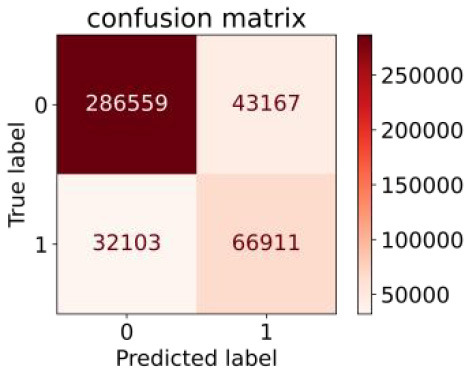	0.676	0.869	0.608	0.899	0.824	0.883
	ADABoost	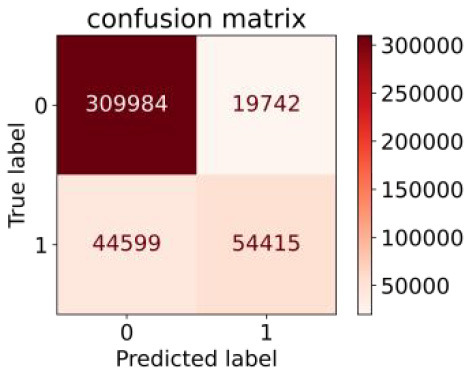	0.550	0.940	0.734	0.874	0.850	0.892
	XGBoost	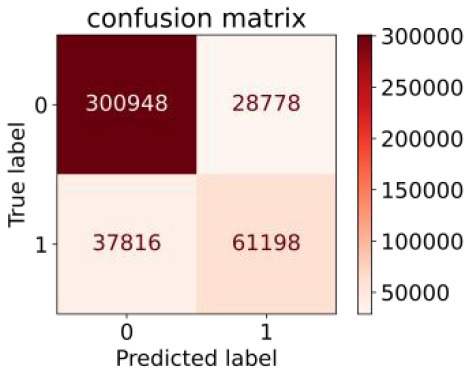	0.618	0.913	0.680	0.888	0.845	0.893
Other methods–based ML models	ANN	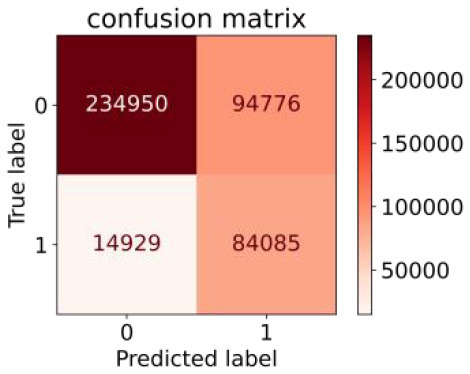	0.849	0.713	0.470	0.940	0.744	0.859
	NB	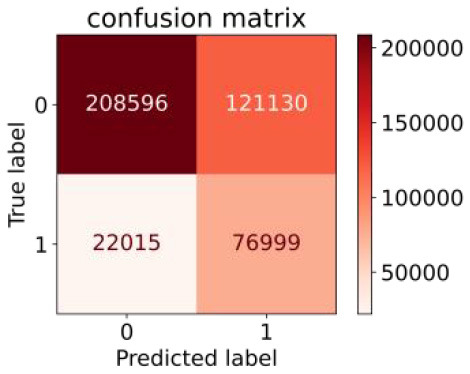	0.778	0.633	0.389	0.905	0.666	0.765
Classic Model	LR	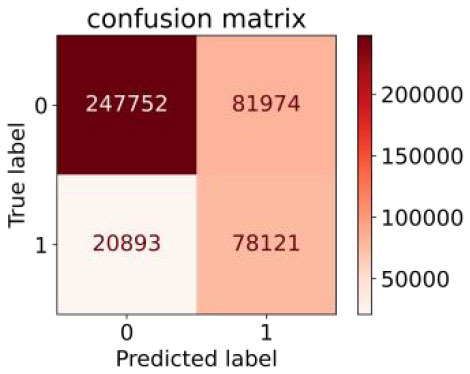	0.789	0.751	0.488	0.922	0.760	0.848

ML, machine learning; CART, classification and regression tree; RF, random forest; ADABoost, adaboost with decision tree; XGBoost, extreme gradient boosting decision tree; ANN, artificial neural network; NB, naive Bayes; LR, logistic regression; AUC, the area under the receiver operating characteristic curve.

**Table 6:** Performance of each algorithm in the test set for semi–laboratory analysis (*n* = 428,740).

**Models**	**Sub–** **Algorithms**	**Confusion matrix**	**Sensitivity**	**Specificity**	**PPV**	**NPV**	**Accuracy**	**AUC**
Tree–based ML models	CART	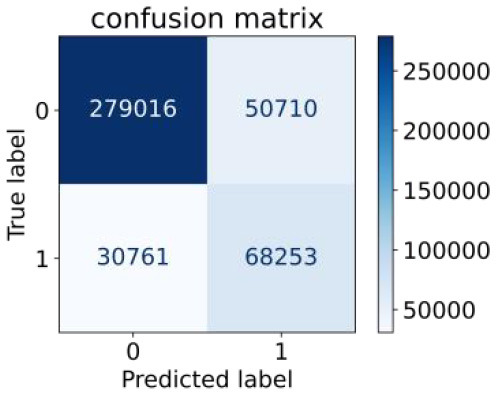	0.689	0.846	0.574	0.901	0.810	0.850
	RF	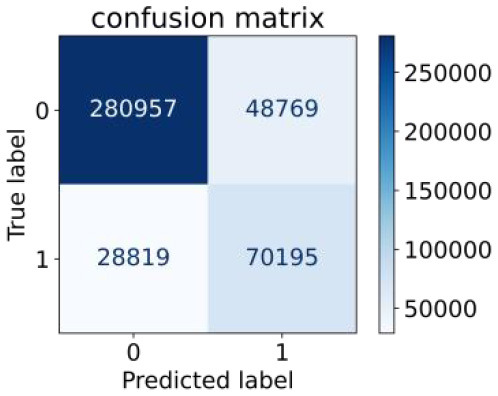	0.709	0.852	0.590	0.907	0.819	0.885
	ADABoost	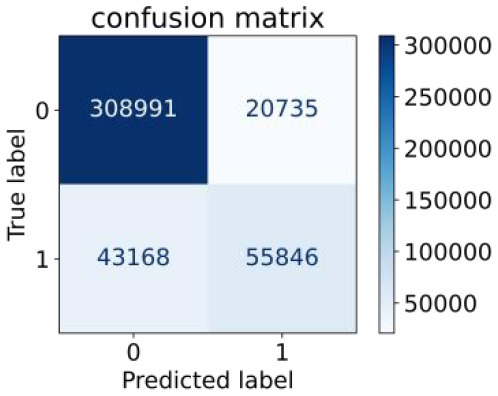	0.564	0.937	0.729	0.877	0.850	0.893
	XGBoost	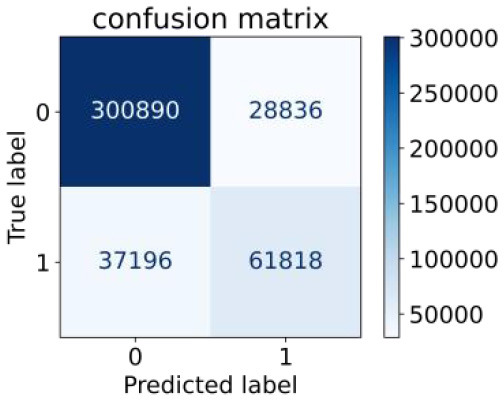	0.624	0.913	0.682	0.890	0.846	0.894
Other methods–based ML models	ANN	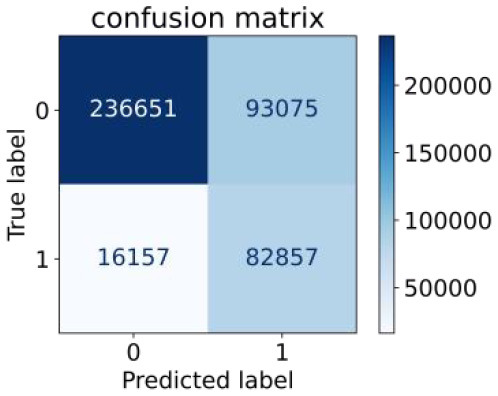	0.837	0.718	0.471	0.936	0.745	0.858
	NB	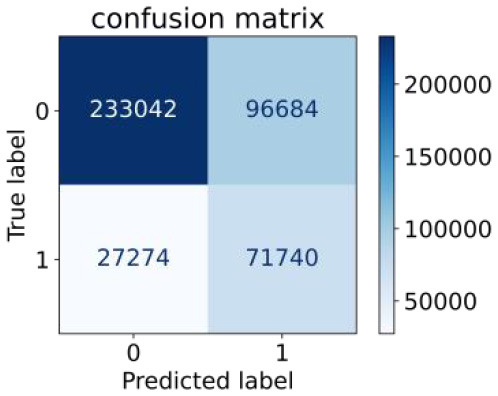	0.725	0.707	0.426	0.895	0.711	0.763
Classic Model	LR	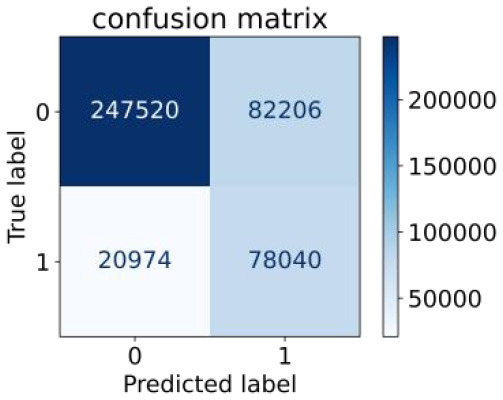	0.788	0.751	0.487	0.922	0.759	0.846

ML, machine learning; CART, classification and regression tree; RF, random forest; ADABoost, adaboost with decision tree; XGBoost, extreme gradient boosting decision tree; ANN, artificial neural network; NB, naive Bayes; LR, logistic regression; AUC, the area under the receiver operating characteristic curve.

### Importance of features

**Figure 6:**
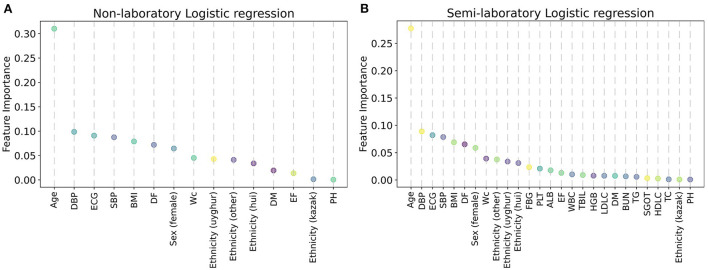
Variable importance of the predictors for the predictive models for hypertension, using logistic regression. **(A)** Non-laboratory model. **(B)** Semi-laboratory model. The variable importance was calculated using the absolute z-statistic of each predictor. SBP, systolic blood pressure; WC, waist circumference; DBP, diastolic blood pressure; ALB, albumin; DF, drinking frequency; ECG, electrocardiogram; BMI, body mass index; EF, exercise frequency; DM, diabetes mellitus; TBIL, total bilirubin; SGOT, serum glutamic-oxaloacetic transaminase; TG, triglyceride; HGB, hemoglobin; PH, parental hypertension; WBC, white blood cell; FBG, fasting blood glucose; HDLC, high density lipoprotein cholesterol; TC, total cholesterol; LDLC, low density lipoprotein cholesterol; BUN, blood urea nitrogen; PLT, platelet; Sex (female) and Sex (male) are dummy variables of sex; ethnicity (uyghur), ethnicity (hui), ethnicity (other), and ethnicity (kazak) are dummy variables of ethnicity.

In this study, the importance of each feature was ranked by the LR model (Figure 6), and it was found that age, DBP, ECG, SBP, BMI, DF, sex (female), WC, ethnicity (uyghur, hui, and other), and FBG were the factors that had a greater impact on hypertension. Afterward, feature importance ranking was conducted for the ML algorithms which performed best in the non-laboratory analyses and semi-laboratory analyses.

In conclusion, considering the results of LR and XGBoost, age, SBP, WC, DBP, ALB, DF, ECG, ethnicity (uyghur, hui, and other), BMI, sex (female), EF, DM, TBIL, and FBG were identified as important factors of hypertension.

Finally, the algorithm architecture proposed in the paper is shown in **Figure 8**. We have constructed the optimal XGBoost algorithm based on non-laboratory and semi-laboratory influencing factors to achieve the prediction of hypertension prevalence in a large-scale population in Xinjiang.

**Figure 7:**
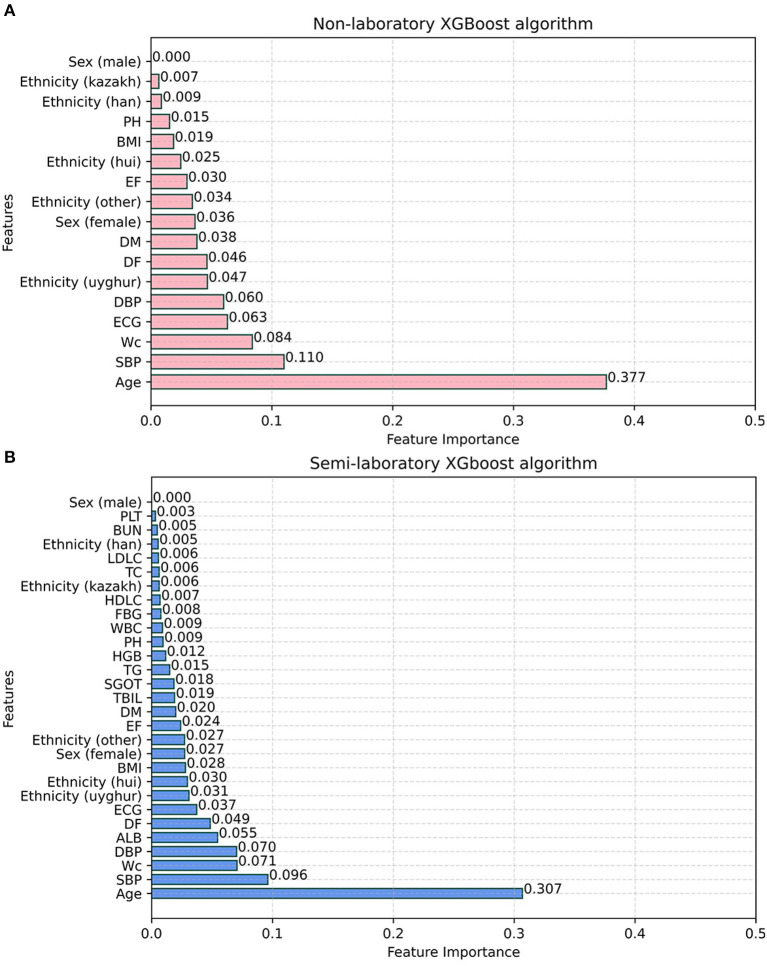
Feature importance of XGBoost algorithm. **(A)** Non-laboratory model. **(B)** Semi-laboratory model. SBP, systolic blood pressure; WC, waist circumference; DBP, diastolic blood pressure; ALB, albumin; DF, drinking frequency; ECG, electrocardiogram; BMI, body mass index; EF, exercise frequency; DM, diabetes mellitus; TBIL, total bilirubin; SGOT, serum glutamic-oxaloacetic transaminase; TG, triglyceride; HGB, hemoglobin; PH, parental hypertension; WBC, white blood cell; FBG, fasting blood glucose; HDLC, high density lipoprotein cholesterol; TC, total cholesterol; LDLC, low density lipoprotein cholesterol; BUN, blood urea nitrogen; PLT, platelet; Sex (female) and Sex (male) are dummy variables of sex; ethnicity (uyghur), ethnicity (hui), ethnicity (other), and ethnicity (kazak) are dummy variables of ethnicity.

XGBoost provides three ways to calculate the importance of each feature, and “gain” was chosen as the calculation method of feature contribution, because it could easily find the most direct features. It was found that age, SBP, WC, ECG, DBP, ethnicity (uyghur and other nationalities), DF, DM, and sex (female) were identified as the top 10 most important factors in the non-laboratory analyses with XGBoost algorithms, while age, SBP, WC, DBP, ALB, DF, ECG, ethnicity (uyghur, hui, and other nationalities), BMI, sex (female), EF, DM, and TBIL were identified as the top 15 most important features in the semi-laboratory analyses with the XGBoost algorithms (Figure 7).

**Figure 8:**
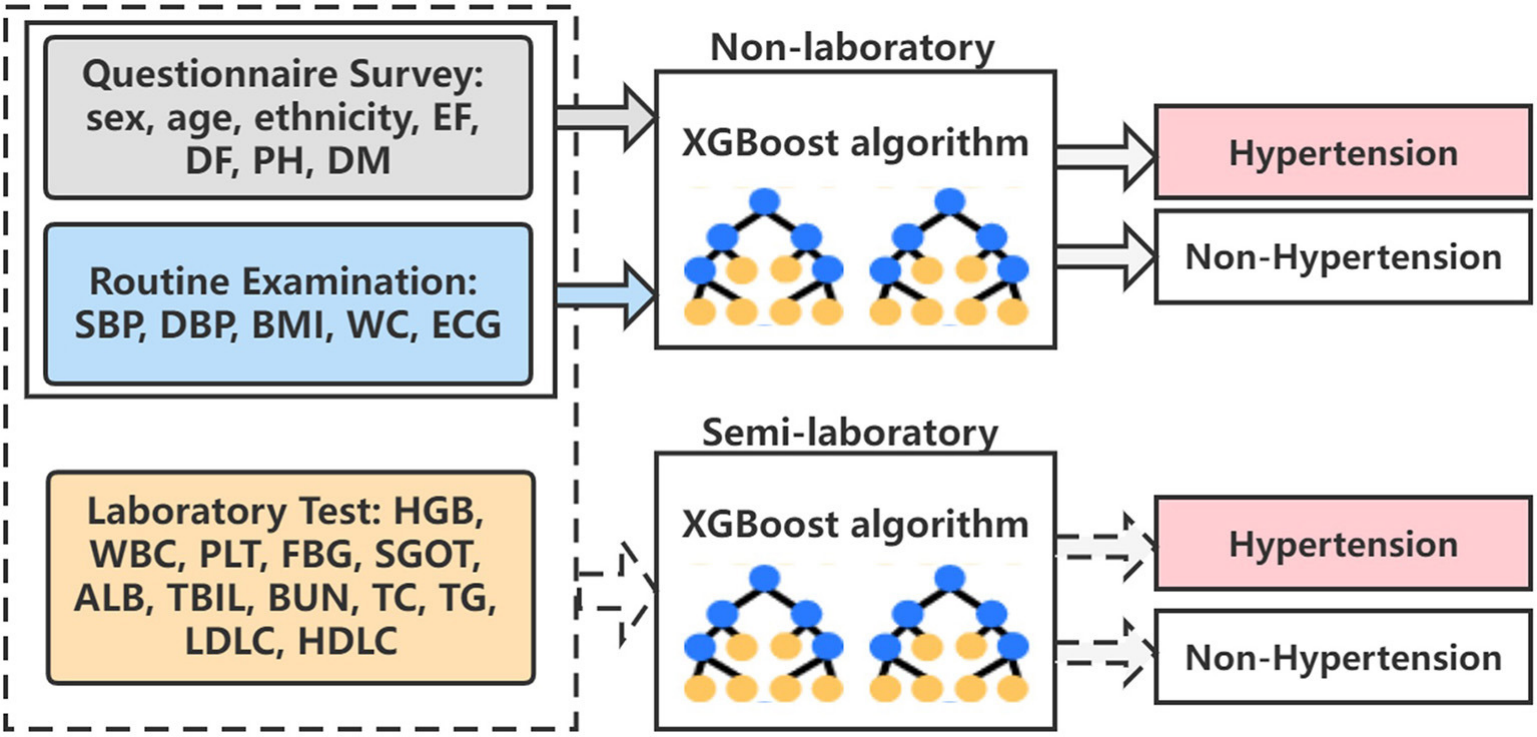
The overall algorithm architecture diagram. EF, exercise frequency; DF, drinking frequency; PH, parental hypertension; DM, diabetes mellitus; SBP, systolic blood pressure; DBP, diastolic blood pressure; BMI, body mass index; WC, waist circumference; ECG, electrocardiogram; HGB, hemoglobin; WBC, white blood cell; PLT, platelet; FBG, fasting blood glucose; SGOT, serum glutamic-oxaloacetic transaminase; ALB, albumin; TBIL, total bilirubin; BUN, blood urea nitrogen; TC, total cholesterol; TG, triglyceride; LDLC, low density lipoprotein cholesterol; HDLC, high density lipoprotein cholesterol.

## Discussion

Between 2012 and 2015, the prevalence of hypertension in China was increasing to a high level (46.4%) according to the 2017 American College of Cardiology/American Heart Association guidelines (38). However, the control and treatment of hypertension are not perfect enough, and people's awareness regarding hypertension was lacking (39). Identifying these potential hypertension patients and initiating appropriate treatment are of priority. In this study, we incorporated 4,287,407 adults who had national physical examinations for non-laboratory and semi-laboratory analyses, respectively, and figured out an optimal prediction of hypertension risk in a large Chinese population by comparing tree-based ML models (CART, RF, ADABoost, and XGBoost), other ML models (NB and ANN), and traditional LR models.

Hypertension is a significant public health issue. The ability to predict the risk of developing hypertension could contribute to disease prevention strategies. At present, many models for hypertension have been established, which show good predicting results. However, these models are limited to a specific population (40–44) or disease (45–47). For example, Xu Y et al. (41) established a prediction model for hypertension in the Xinjiang kazak population by using 14 predictors, including age, smoking, alcohol consumption, baseline BMI, baseline DBP, baseline SBP, daily salt intake, and yak butter intake. Kanegae H et al. (43) developed a high-precision prediction model for hypertension based on artificial intelligence by incorporating age, BMI, WC, SDP, DBP, Cardio-Ankle vascular index, uric acid, and other factors. Qi H et al. (45) established a micro-RNA screening and prediction model for salt-sensitive hypertension at the miRNA molecular level. Factors unique to these studies may be the main reason why the model achieves good predictive results in different populations. The classic hypertension prediction model Framingham Risk Score (FRS) (7) believes that age, sex, SDP, DBP, BMI, PH, and smoking are important influencing factors of hypertension. FRS has been verified in European population studies and has shown good differentiation and calibration (9). Carson AP et al. (40) also applied it to the prediction and assessment of hypertension risk in young people and achieved good results. However, a study about the FRS model indicated that it is not suitable for the Chinese population (11). Therefore, this study included ethnicity, WC, EF, ECG, DM, and other characteristics based on FRS, which gained good predicting results.

It has been widely confirmed that the prevalence of hypertension in different genders was diverse (48–51). This study showed that differences existed between the two genders. In the age groups of 18–29 and 30–45, the prevalence of hypertension in men was significantly higher than in women, while in the 46–65 and over 65 age groups, an opposite trend was observed. This difference might be due to hormonal differences or lifestyle differences (50, 52). Studies have shown that the blood pressure of premenopausal women was often lower than that of men of the same age. After menopause, the prevalence of hypertension in women gradually increased, and after the age of 65, the prevalence of hypertension in women was significantly higher than in men (51, 53). The above findings indicated that there were gender differences in the underlying pathological mechanisms of hypertension.

Previous studies have demonstrated differences in the prevalence of hypertension between different ethnicities (54–57) and confirmed that ethnicity could be a predictor of hypertension (58). Therefore, ethnicity was incorporated into the prediction model, and the results also indicated that it could be an important predictor of hypertension in the Chinese population, especially in uygur, hui, and other nationalities.

According to the World Health Organization (WHO), the global increase in the prevalence of hypertension has been attributed to persistent stress, excess weight, physical inactivity, harmful alcohol consumption, and an unhealthy diet (59). Also, our model also proved that WC, BMI, EF, and DF are important influencing factors of hypertension. Our findings also suggest that ECG was an important predictor of hypertension, which was consistent with other studies (60–62). The pathogenesis of DM and hypertension mutually promote and influence each other (63–65), which makes the prediction models have a general limitation and may not be applicable to the DM population (7, 13, 66). In order to avoid this deficiency, this important factor was considered in the inclusion of risk factors and was included as a predictor of hypertension, and the results also showed its important role in predicting hypertension. The semi-laboratory analyses of this study showed that ALB levels were important influencing features of hypertension. Hypertension is associated with endothelial dysfunction, insulin resistance, inflammation, and oxidative stress (67, 68), while ALB has anti-inflammatory and antioxidant effects (69). The study by Oda E et al. (70) also showed the same findings about ALB as our study. A report published by Nilsson PM in 2019 showed that after multiple adjustments for age, sex, body mass index, smoking, drinking habits, dyslipidemia, chronic kidney disease, and blood uric acid, fasting blood glucose at a high baseline level was an independent risk marker for new-onset hypertension. Afterward, TatSumi et al. (71) showed that fasting blood glucose was a good predictor of hypertension through a 5-year cohort study, which was consistent with our findings.

This study implied that the semi-laboratory analyses incorporating blood test indicators did not show a significant improvement in predictive performance compared to the non-laboratory analyses. The feature importance ranking plot of the XGBoost algorithm also showed that the blood test factors were not very important for the identification of hypertension.

There are several advantages to this study. First, this study was based on a large amount of population data in China, which was highly generalizable and representative. In addition, our dataset included multiple major ethnic groups in China, which better assessed the characteristics of the Chinese population. Besides, we carried out both non-laboratory analyses and semi-laboratory analyses, respectively, and found two optimal models that were suitable for people in different regions. Especially, in non-laboratory analyses, simple and easily available variables were used to build a predictive model with high performance, which saves blood testing and extra manpower, as well as greatly promotes the diagnosis and screening of hypertension in economically underdeveloped remote areas (15). We obtained a satisfied predictive effect of our models, for example, the AUC values of XGBoost were 0.893 and 0.894, respectively. As far as we know, the effects of our model were better than most of the known models, which might be due to the fact that the model was built on many features and included a big sample.

There are several limitations to this study. First, the causal relationship cannot be analyzed from the cross-sectional data of the health screening component, which needs to be further verified in future studies. Second, the data of this study were based on the physical examination data of residents in the Xinjiang region of China, which may limit the extrapolation of results. Third, EF, DF, and DM are all based on a questionnaire survey, and participants reported themselves through recall, which can lead to memory errors. Considering privacy and other reasons, participants failed to truthfully fill in their DM status, so the prevalence of diabetes was underestimated. Finally, in the current study, only self-reported parental history of hypertension was available. A previous study indicated that children's self-reported parental history of hypertension had a high positive predictive value but a low negative predictive value, suggesting that more participants may classify their parents as normotensive while their parents were actually hypertensive (72).

## Conclusion

In summary, on the basis of a cross-sectional study involving 4,287,407 participants, we carried out the non-laboratory and semi-laboratory analyses, by constructing the tree-based ML models, other ML models, and traditional LR model and obtaining the optimal algorithm for predicting the risk of hypertension in a large-scale Chinese population. This study showed that tree-based ML models (XGBoost algorithm) performed excellently in identifying hypertensive patients, while blood test factors had little effect on improving the hypertension prediction model. As we know, this study is the first one to establish non-laboratory and semi-laboratory hypertension prediction models on the basis of multi-ethnic and large samples by systematically and comprehensively comparing various algorithms, which provided a new approach to the prediction and prevention of hypertension.

## Data availability statement

The original contributions presented in the study are included in the article/Supplementary materials, further inquiries can be directed to the corresponding authors.

## Ethics statement

The studies involving human participants were reviewed and approved by this study was conducted in accordance with the principles outlined in the Helsinki Declaration and was approved by the Ethics Committee and Institutional Review Committee of the Xinjiang Uygur Autonomous Region Center for Disease Control and Prevention. The patients/participants provided their written informed consent to participate in this study.

## Author contributions

YiZ and YW conceived the study. WJ and YuZ collected the data. WJ and YC performed the statistical analyses. WJ and YuZ drafted the manuscript. YiZ critically reviewed and edited the manuscript. All authors contributed to data analysis, drafting, and revising of the article, gave final approval of the version to be published, agreed on the journal to which the article has been submitted, and agree to be accountable for all aspects of the work.

## Funding

This work was supported by the National Key Research and Development Program of China [No. 2018YFC0116900], the National Natural Science Foundation of China (NSFC) [No. 61876194], the Key Research and Development Program of Guangdong Province, China [No. 2018B010109006], the Science and Technology Innovation Special Project of Guangdong Province, China [No. 202011020004], and the Natural Science Foundation of Guangdong Province, China [No. 2021A1515011897].

## Conflict of interest

The authors declare that the research was conducted in the absence of any commercial or financial relationships that could be construed as a potential conflict of interest.

## Publisher's note

All claims expressed in this article are solely those of the authors and do not necessarily represent those of their affiliated organizations, or those of the publisher, the editors and the reviewers. Any product that may be evaluated in this article, or claim that may be made by its manufacturer, is not guaranteed or endorsed by the publisher.
