# Supplementary material for: Development and validation of prediction models for hypertension risks: A cross-sectional study based on 4,287,407 participants
Source: Front Cardiovasc Med. 2022 Sep 26;9:928948. doi: 10.3389/fcvm.2022.928948 (PMC9548597; doi:10.3389/fcvm.2022.928948)
Supplement: Supplementary file 1 [file Data_Sheet_1.docx]

**Supplemental Materials**

**Development and validation of prediction models for hypertension risks: a cross-sectional study based on 4,287,407 participants**

Weidong Ji, Yushan Zhang, Yinlin Cheng, Yushan Wang and Yi Zhou

**Table of Contents**

**Figure S1** The receiver operating characteristic curves for each algorithm in the test set for non-laboratory analysis.

**Figure S2** The receiver operating characteristic curves for each algorithm in the test set for semi-laboratory analysis.

**Table S1** Performance of each algorithm in the validation set for non-laboratory analysis.

**Table S2** Performance of each algorithm in the validation set for semi-laboratory analysis.


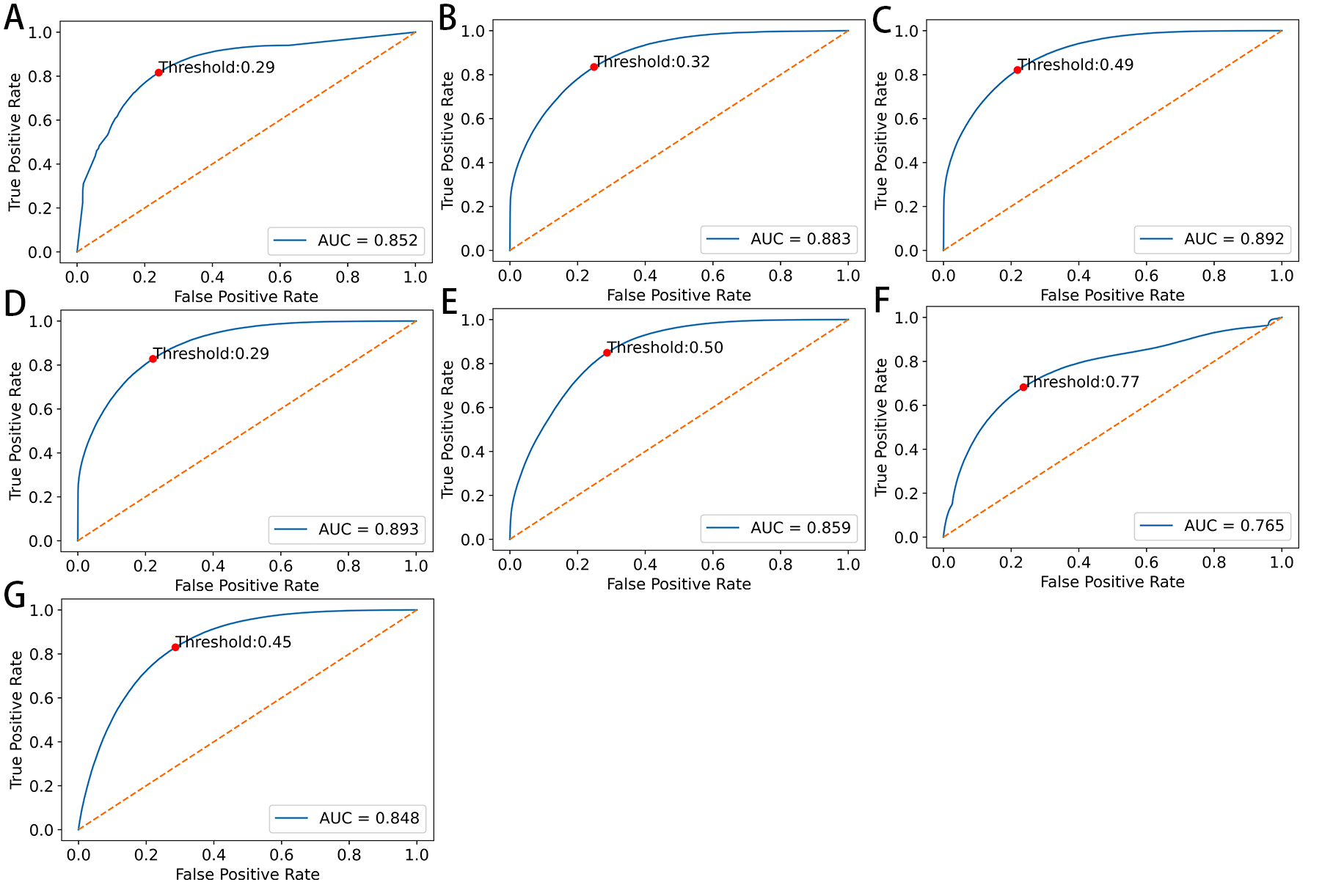


**Figure S1** The receiver operating characteristic curves for each algorithm in the test set for non-laboratory analysis. (A) classification and regression tree. (B) random forest. (C) adaboost with decision tree. (D) extreme gradient boosting decision tree. (E) artificial neural network. (F) naive bayes. (G) logistic regression. Abbreviations: AUC, the area under the receiver operating characteristic curve.


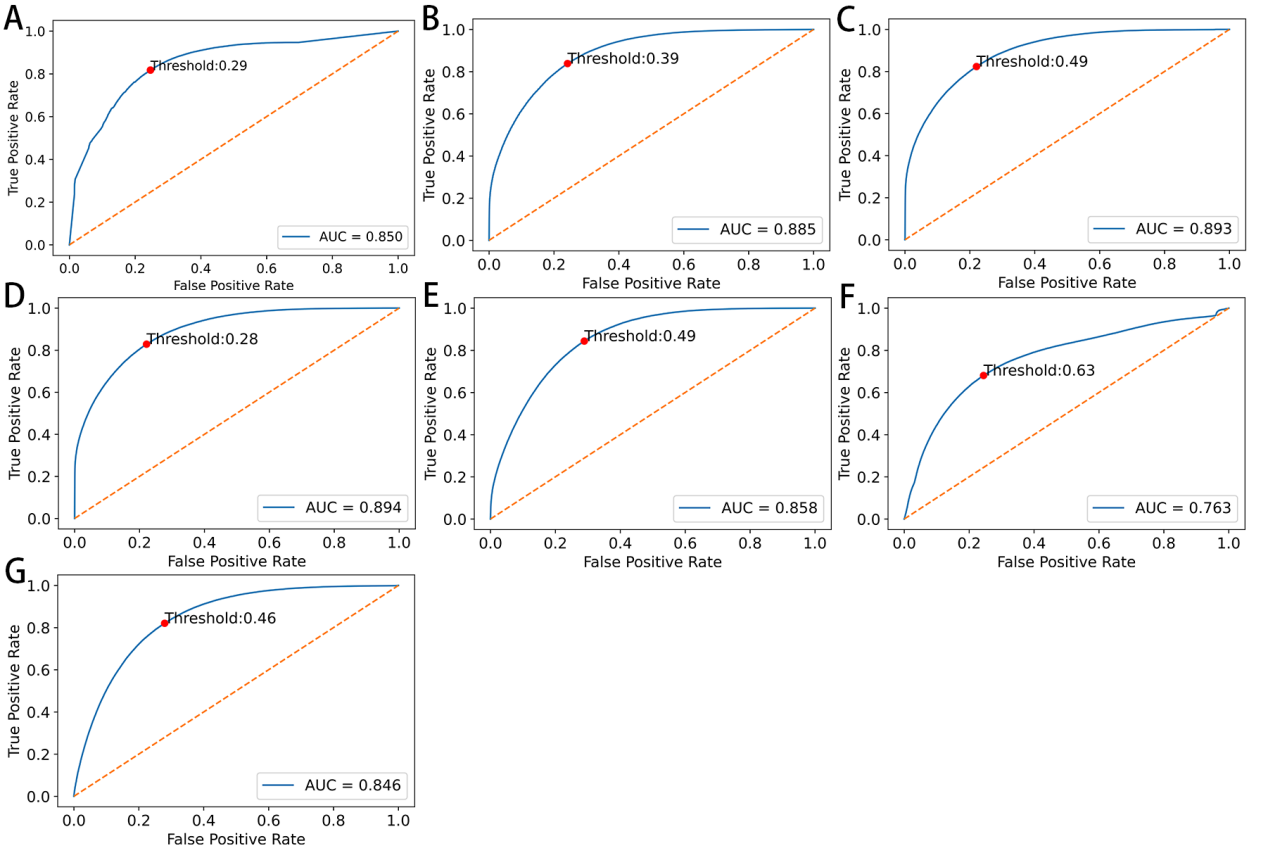


**Figure S2** The receiver operating characteristic curves for each algorithm in the test set for semi-laboratory analysis. (A) classification and regression tree. (B) random forest. (C) adaboost with decision tree. (D) extreme gradient boosting decision tree. (E) artificial neural network. (F) naive bayes. (G) logistic regression. Abbreviations: AUC, the area under the receiver operating characteristic curve.

**Table S1** Performance of each algorithm in the validation set for non-laboratory analysis (n=857,482)

| Models | Sub-Algorithms | Confusion matrix | Sensitivity | Specificity | PPV | NPV | Accuracy | AUC |
| --- | --- | --- | --- | --- | --- | --- | --- | --- |
| Tree-based ML models | CART | 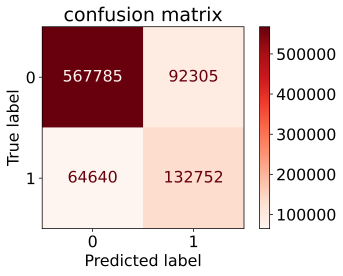 | 0.673 | 0.860 | 0.590 | 0.898 | 0.817 | 0.852 |
|  | RF | 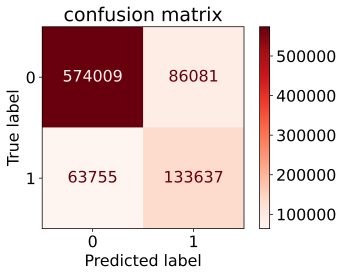 | 0.677 | 0.870 | 0608 | 0.900 | 0.825 | 0.883 |
|  | ADABoost | 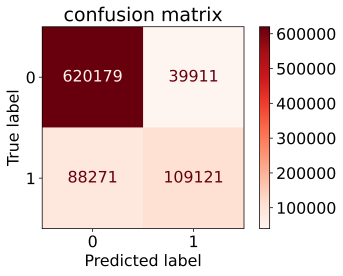 | 0.553 | 0.940 | 0.732 | 0.875 | 0.851 | 0.893 |
|  | XGBoost | 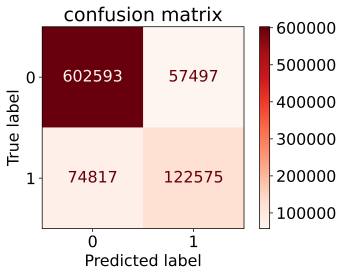 | 0.621 | 0.913 | 0.681 | 0.890 | 0.846 | 0.894 |
| Other methods-based ML models | ANN | 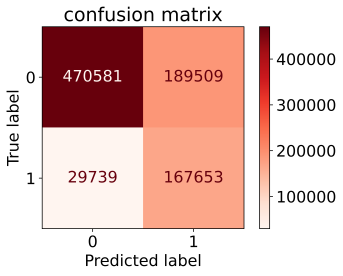 | 0.849 | 0.713 | 0.469 | 0.941 | 0.744 | 0.860 |
|  | NB | 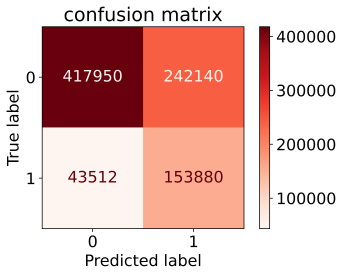 | 0.780 | 0.633 | 0.389 | 0.906 | 0.667 | 0.766 |
| Classic Model | LR | 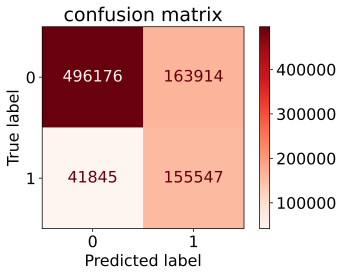 | 0.787 | 0.752 | 0.487 | 0.922 | 0.760 | 0.848 |

**Abbreviations:** ML, machine learning; CART, classification and regression tree; RF, random forest; ADABoost, adaboost with decision tree; XGBoost, extreme gradient boosting decision tree; ANN, artificial neural network; NB, naive bayes; LR, logistic regression; AUC, the area under the receiver operating characteristic curve.

**Table S2** Performance of each algorithm in the validation set for semi-laboratory analysis (n=857,482)

| Models | Sub-Algorithms | Confusion matrix | Sensitivity | Specificity | PPV | NPV | Accuracy | AUC |
| --- | --- | --- | --- | --- | --- | --- | --- | --- |
| Tree-based ML models | CART | 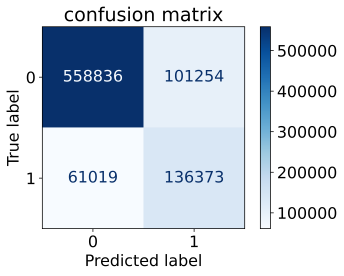 | 0.691 | 0.847 | 0.574 | 0.902 | 0.811 | 0.851 |
|  | RF | 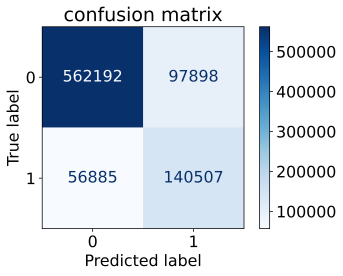 | 0.712 | 0.852 | 0.589 | 0.908 | 0.819 | 0.886 |
|  | ADABoost | 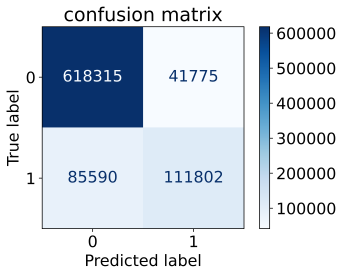 | 0.566 | 0.937 | 0.728 | 0.878 | 0.851 | 0.893 |
|  | XGBoost | 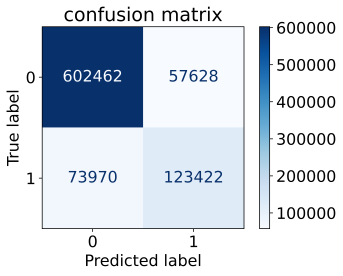 | 0.625 | 0.913 | 0.682 | 0.891 | 0.847 | 0.895 |
| Other methods-based ML models | ANN | 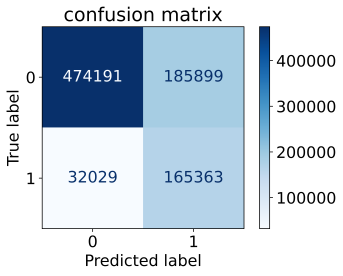 | 0.838 | 0.838 | 0.471 | 0.937 | 0.746 | 0.858 |
|  | NB | 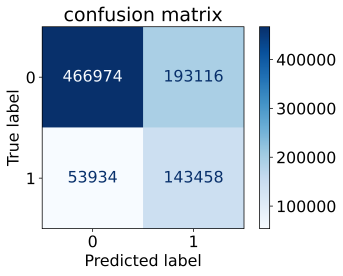 | 0.727 | 0.707 | 0.426 | 0.896 | 0.712 | 0.764 |
| Classic Model | LR | 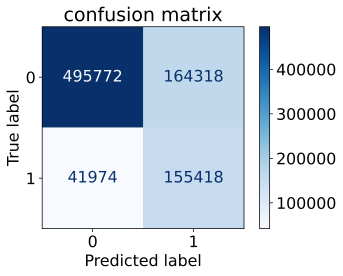 | 0.787 | 0.751 | 0.486 | 0.922 | 0.759 | 0.846 |

**Abbreviations:** ML, machine learning; CART, classification and regression tree; RF, random forest; ADABoost, adaboost with decision tree; XGBoost, extreme gradient boosting decision tree; ANN, artificial neural network; NB, naive bayes; LR, logistic regression; AUC, the area under the receiver operating characteristic curve.
